# Intact architectures of myophage phi92 in extended and contracted states

**DOI:** 10.1371/journal.ppat.1014494

**Published:** 2026-08-07

**Authors:** Yuan Chen, Yuning Peng, Yuanyuan Liu, Yewei Zhang, Hao Xiao, Wenyuan Chen, Binning Sun, Jianxun He, Xiaorong Yang, Jing Zheng, Hongrong Liu

**Affiliations:** 1 Institute of Interdisciplinary Studies, Key Laboratory for Matter Microstructure and Function of Hunan Province, Key Laboratory of Low-dimensional Quantum Structures and Quantum Control, School of Physics and Electronics, Hunan Normal University, Changsha, China; 2 Hunan Research Center of the Basic Discipline for Quantum Effects and Quantum Technologies, Hunan Normal University, Changsha, China; Yale University, UNITED STATES OF AMERICA

## Abstract

Since conventional antibiotics frequently fail to effectively treat infections caused by encapsulated bacteria, phage therapy has gained attention as a potential treatment approach. However, the understanding of phages that can specifically infect encapsulated bacteria—particularly myophages—remains limited, especially regarding their structures with multi-states, and infection and contraction mechanisms, such as tail fiber conformational changes and what triggers tail contraction. In this study, we resolved the intact structures of phi92, which possesses a broad host range encompassing both encapsulated and non-encapsulated strains of *Escherichia coli* strains and diverse *Salmonella* strains, in both its extended and contracted states by cryo-electron microscopy (cryo-EM). We identified and built atomic models for most components in the head, connector, tail, and baseplate. Notably, we inferred that one of the three fibers corresponds to fiber I (gp143) and identified another as fiber III (gp147). We propose that fiber I specifically degrades host capsular polysaccharides, while fiber III mediates stable adsorption to the host cell membrane. Phi92 achieves broad host adaptability through its multiple fibers, thereby conferring a significant competitive advantage when infecting bacteria with distinct types. Comparison of the two states reveals that significant conformational rearrangements of fiber III and baseplate periphery play a pivotal role in triggering sheath contraction. This study elucidates the trigger mechanism of the contractile nanomachine in phi92-like myophages with a baseplate architecture, providing a crucial structural foundation for developing myophage-based therapies against encapsulated, drug-resistant bacteria.

## Author summary

With the increasing severity of the problem of drug-resistant bacteria, phage therapy is regaining renewed attention as an effective alternative strategy. Myophage phi92 exhibits broad-spectrum infectivity against multiple clinically prevalent and resistance-prone bacterial pathogens, including both encapsulated and non-encapsulated strains of *E. coli*, as well as diverse *Salmonella strains*. Therefore, phi92 represents a promising candidate for combating multidrug-resistant bacterial infections. However, the molecular mechanisms underlying its ability to penetrate the capsular barrier and trigger tail contraction remain poorly understood. Here, we reported the near-complete structures of phi92 in its extended and contracted states. We inferred fiber I (gp143) and identified fiber III (gp147), which function as an enzyme to degrade host capsular polysaccharides and as the mediator of irreversible binding to the host outer membrane, respectively. This multi-fiber system is essential for infecting encapsulated bacteria. Structural comparison of the two states reveals that significant conformational rearrangements in the fibers and baseplate are critical for triggering tail contraction and subsequent genome injection. This study reveals the molecular details of a universal mechanism among phi92-like phages by which conformational changes in baseplates trigger tail contraction, providing structural insights for engineering or redesigning phages to overcome bacterial resistance.

## Introduction

Encapsulated bacteria have a polysaccharide capsule outside the single membrane layer (Gram-positive) or the double membrane layers (Gram-negative) [1]. This capsule not only shields the bacteria from nonspecific host defenses but also serves as a source of nutrients [2]. With the widespread dissemination of antibiotic-resistant bacteria, particularly encapsulated pathogenic bacteria, the clinical efficacy of conventional antibiotics is facing severe challenges [3,4]. As a specifically targeted and highly efficient alternative to antibiotics, bacteriophages (phages) are emerging as a promising therapeutic approach for the prevention and control of bacterial infections [5,6]. The majority of phages are tailed phages, classified into three morphological groups: myophages with a contractile tail, podophages with a short non-contractile tail, and siphophages with a long, flexible non-contractile tail [7]. However, a limited understanding of the mechanisms underlying phage infection and genome delivery has significantly constrained their clinical translation and potential for rational engineering. Given that bacterial capsules can impede antibiotic penetration and facilitate immune evasion, conventional antibiotics often exhibit suboptimal efficacy against encapsulated pathogens [2]. Consequently, in-depth investigations into phage lineages capable of efficiently breaching such barriers are critically important. Among various phages, myophages may exhibit inherent advantages against drug-resistant bacteria surrounded by capsules or embedded within biofilms, owing to their unique contractile tails [8]. For example, phi92 is a lytic myophage [9] that specifically infects encapsulated *Escherichia coli*, highlighting its therapeutic potential against drug-resistant bacterial infections. Structurally, myophages consist of an icosahedral head, a portal-connector, and a complex tail. The tail contains a contractile sheath surrounding a rigid inner tail tube, which terminates in a complex baseplate equipped with lateral fibers that mediate host recognition and attachment [10,11]. Some myophages have evolved a synergistic invasion strategy by using a contractile sheath in conjunction with tail fibers [10,12] that carry capsule depolymerase activity. This strategy involves the enzymatic degradation of extracellular polysaccharide barriers, such as capsules, coupled with the mechanical puncturing of the tail driven by sheath contraction [13,14], thereby enabling efficient breaching of membrane defenses. However, recent high-resolution structural studies of myophages have focused predominantly on the extended state or the contracted state lacking the baseplate [11,15–21]. To date, only two phages, T4 [22] and E217 [23], have been resolved at high resolution in two states with an intact baseplate. Although the structures of contractile injection systems (CISs), evolutionarily related to myophages tails, have provided important insights into tail contraction, CISs rely on a relatively simple physical injection mechanism, which is different from the intricate mechanisms deployed by myophages for host recognition, infection, and DNA delivery. For myophages that specifically infect encapsulated bacteria, the molecular mechanisms underlying host recognition, tail sheath contraction, and dynamic conformational changes remain poorly understood. This lack of structural information significantly hinders the rational design, engineering, and clinical development of these highly effective phages.

The myophage phi92 was first identified in 1983 from pathogenic *E. coli* strains encapsulated in a polysialic acid layer [24]. Notably, phi92 exhibits an exceptional ability to infect a broad range of clinically prevalent bacterial pathogens that are highly prone to developing drug resistance. Its host range includes both encapsulated and non-encapsulated strains of *E. coli*, as well as multiple pathogenic *Salmonella* strains [9]. Owing to this property, phi92 serves as a valuable research model and a potential therapeutic candidate for phage therapy against multidrug-resistant and especially encapsulated bacteria. Additionally, phi92 demonstrates notable translational value in bioindustry and its protein gp150 has been identified as a colanic acid-degrading enzyme (CAE) that specifically hydrolyzes the β-1,4 glycosidic linkage between glucose and fucose [25], and has been used to produce colanic acid oligosaccharides (CAOSs) with broad industrial applications [25]. The intact phi92 virion comprises an icosahedral head, a portal-connector, a contractile tail and a baseplate flanked laterally by multiple sets of tail fibers [9]. Although previous studies have reported the cryo-electron microscopy structure of mature phi92 [26], for which no corresponding maps or coordinates have yet been deposited in the Protein Data Bank (PDB) or the Electron Microscopy Data Bank (EMDB), as well as the crystal structures of spike proteins gp138 [27] and fiber protein gp143 [12], critical gaps in our structural understanding of this phage remain. For example, although the gp143 protein of phi92 functions as an endosialidase enzyme that specifically degrades host capsular polysaccharide [9], its location within the phage particle is unconfirmed. Significantly, it remains unclear how the baseplate of phi92 enables penetration of the complex bacterial capsular barrier and triggers tail contraction, due to the lack of structural information on its contracted state. Genomic analysis demonstrates that phi92 shares numerous protein homologs with phages rv5 [28] and PVP-SE1 [29]. Notably, the complex tail structure of PVP-SE1, composed of at least five types of proteins, resembles that of phi92. The contraction and infection mechanisms of myophages with multiple tail fibers remain to be elucidated. Therefore, a detailed mechanistic understanding of the infection and contraction processes of phi92 and phi92-like myophages will provide indispensable structural biological insights for the future design and engineering of highly efficient and precisely targeted phage therapies.

In myophages, the molecular mechanisms of tail contraction and bacterial outer membrane penetration, especially for phages infecting encapsulated bacteria, are not fully understood. In this study, we resolved the high-resolution structures of the myophage phi92 in both native extended and urea-induced contracted states using cryo-EM. By combining cryo-EM maps with AlphaFold3 predictions [30], we identified nearly all protein components of the head (gp123 and gp124), the portal-connector (gp120, gp126, gp128, and gp129), the tail (gp130 and gp131), and the baseplate (gp134, gp135, gp136, gp137, gp138, gp139, gp145, and gp146), along with fiber I (gp143), and fiber III (gp147). Structural comparisons of phi92 in both extended and contracted states reveal that the portal-connector and the tail tube remain unchanged, except for the C-terminus of the tail terminator protein, which extends to accommodate sheath contraction. Fiber III, the baseplate wedge, and the sheath initiator protein undergo a series of significant conformational changes, leading to wedge expansion, sheath compaction and contraction, and exposure of ~460 Å of the rigid tail tube. Analysis of phi92’s conformational dynamics provides insights into the infection and contraction mechanisms of myophages that infect encapsulated bacteria. Structural and functional characterization of phi92’s multiple tail fibers not only offers potential for developing industrially useful enzymes, such as polysaccharide depolymerases, but also provides a framework for designing new therapeutic approaches against antibiotic-resistant bacteria, including engineered phage cocktails and phage-derived enzyme and antibiotics.

## Results

### Overall structures of phi92 in its two distinct states

**Fig 1:**
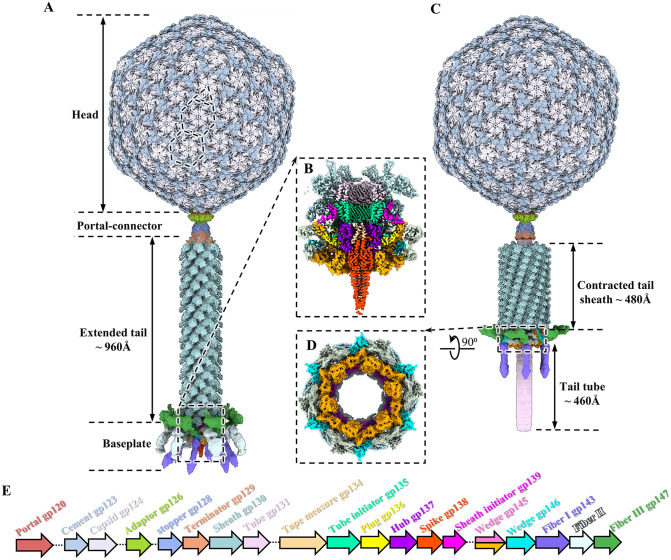
Overall structures of myophage phi92 with its extended and contracted states. **(A)** Side view of the intact structure of extended phi92. **(B)** Cut-open view of the density map of the baseplate in the extended phi92. **(C)** Side view of the intact structure of contracted phi92. **(D)** Bottom view of the density map of the baseplate in the contracted phi92. **(E)** Organization of phi92 genome segments. The color code is applied to panels A-D.

For the collection of cryo-EM data, phage phi92 was purified from the *E.coli* BL21 strain (S1A Fig) and contracted phi92 was produced by treating extended phi92 particles with 3M urea (S1B Fig). A total of 32,356 extended and 9,796 contracted particles were extracted from the native sample and the urea-treated sample, respectively. Icosahedral reconstruction method [31] was utilized to obtain an icosahedral head structure of the extended phi92 at a resolution of 3.9 Å. Local structures of the 5-fold region of the icosahedral head, capsid-portal, portal-adaptor, and stopper-tail of extended phi92 were reconstructed to the resolutions of 3 Å, 4.5 Å, 3.3 Å, and 3.5 Å, respectively, by combining the symmetry-mismatch reconstruction [32,33] and the local reconstruction methods [34,35] (S1C Fig). The baseplate structure of the extended phi92 was resolved to a resolution of 3.2 Å using RELION software [36] (S1C Fig). Local resolution maps for the extended phi92 are shown in S2 Fig. The high-resolution density map allowed us to build atomic models for the following extended phi92 components (Figs 1A and S3): major capsid protein (MCP, gp124), cement protein (CP, gp123), portal protein (gp120), adaptor protein (gp126), stopper protein (gp128), tail terminator protein (gp129), tail sheath protein (gp130), tail tube protein (gp131), tube initiator protein (gp135), sheath initiator protein (gp139), C-terminal region of the tape measure protein (TMP, gp134), hub protein (gp137), spike protein (gp138), plug protein (gp136), and heterotrimeric wedge complex (gp145-gp146). The N-terminus of TMP gp134 and three sets of tail fibers were not resolved to near-atomic resolution, likely due to their flexibility. The structural resolution was estimated using the Fourier shell correlation criterion with a cut-off of 0.143 according to the “gold standard” method [37].

In the extended state, the icosahedral head of phi92 is composed of 775 copies of the MCP gp124 with a triangulation number T = 13. The 780 copies of the CP gp123 form 260 trimers attached to the icosahedral three-fold and quasi-three-fold axes on the head surface (Fig 1A). The head-to-tail connector (Fig 1A), anchored at a unique five-fold vertex of the head by a dodecameric portal (gp120), is composed of a dodecameric adaptor (gp126), a hexameric stopper (gp128), and a hexameric tail terminator (gp129). The tail consists of 24 hexameric stacked rings of tube (gp131) and sheath (gp130) (Fig 1A, 1B). The baseplate contains seven protein components, including a trimeric hub gp137, a trimeric spike gp138, a trimeric TMP gp134, a hexameric tube initiator gp135, a six-fold symmetric sheath initiator gp139, six heterotrimeric wedges gp145-gp146, and a six-fold symmetric plug gp136 (Fig 1B). Three sets of tail fibers are attached to the wedge of the baseplate (Fig 1A).

The same methods were used to reconstruct the structures of the head, portal-adaptor, stopper-terminator, tail sheath, and baseplate of the contracted phi92 to resolutions of 3.6 Å, 4.4 Å, 4.9 Å, 3.7 Å, and 4.2 Å, respectively (S1C Fig). Local resolution maps of the contracted phi92 are shown in S4 Fig. The contracted connector was resolved only at medium resolution, likely due to destabilization during centrifugation. Urea induces the detachment of the baseplate tip during tail contraction, resulting in the absence of detectable density (Fig 1C). Additionally, the contracted baseplate periphery remains anchored to the tail sheath and retains six-fold symmetry (Fig 1C, 1D). Using the atomic models of the extended phi92 as references, we built models for the head (gp123, gp124), the portal-connector (gp120, gp126, gp128, gp129), the tail (gp130), and the baseplate (gp139, gp145, gp146) (S5 Fig). Compared with extended phi92, contracted phi92 undergoes dramatic structural rearrangements that facilitate DNA and TMP ejection, baseplate and tail sheath contraction toward the connector, and exposure of ~460 Å of the rigid tail tube (Fig 1A, 1C). Gene products are listed in Fig 1E. Data collection and reconstruction statistics are detailed in S1 Table.

### Structures of the head, portal, connector, and tail in the extended state

**Fig 2:**
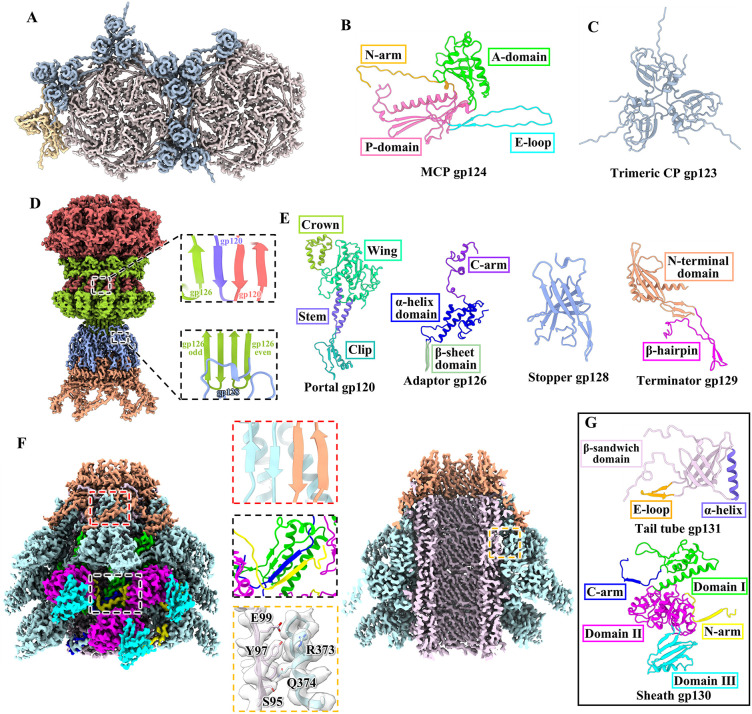
Structures of the head, portal-connector complex, and tail of extended phi92. **(A)** Density map of an asymmetric unit of the icosahedral head corresponding to the dashed area in Fig 1A. One penton monomer and two hexons are colored yellow and light pink, respectively; the cement proteins are colored light blue. **(B)** Ribbon model of the MCP gp124 shown in four domains. **(C)** Ribbon model of the trimeric CP gp123. **(D)** Side view of portal-connector complex (density map). Color codes are identical to that used in Fig 1A. The insets show the zoomed-in views of the interactions between the portal and adaptor (top), and between the adaptor and stopper (bottom). **(E)** Ribbon model of the portal protein gp120, adaptor protein gp126, stopper protein gp128, and terminator protein gp129, colored according to their domains. **(F)** Side (left) and cut-open (right) views of the density maps of the tail (only terminator, three sheath and four tube rings). The color coding is identical to that used in Fig 1A. The insets show the zoomed-in views of the four-stranded β-sheet interactions between the terminator and sheath (top) and between two adjacent sheath rings (middle), and of the cation-π interaction (Y97-R373), hydrogen bond (S95-Q374) and salt bridge (E99-R373) between the tube and sheath (bottom). **(G)** Ribbon models of the tail tube protein gp131 and sheath protein gp130, colored according to their domains.

Based on the density map of the phi92 head, we built the atomic models of MCP gp124 and CP gp123 (Figs 2A-2C and S3B). The MCP gp124 adopts a canonical HK97 fold [38] and can be divided into four domains: N-arm, E-loop, P-domain, and A-domain. The phi92 head comprises 775 copies of the MCP gp124 organized into 11 pentons and 120 hexons (S6A Fig), forming an icosahedral shell with a triangulation number of 13. The trimeric gp123 CP adopts a β-tulip fold located at the quasi-three-fold and three-fold axes of the icosahedral head (Fig 2A, 2C), structurally similar to the CPs of phages T1 [39], lambda [40], and TW1 [41]. The CP gp123 forms tight interactions with the MCP gp124 at both the icosahedral three-fold and quasi-three-fold symmetry axes (S6B Fig). Specifically, the N‑terminus of gp123 protrudes outward to form a four-stranded β-sheet with the E-loop of an underlying gp124 and the N-terminus of a neighboring gp124 from the same capsomer, further enhancing capsid stability (S6B Fig).

The portal, occupying a unique five-fold vertex of the head, contains 12 copies of protein gp120 (Fig 2D) and exhibits high structural similarity to portals from other tailed phages [42]. Each gp120 can be divided into four domains: the crown domain, the wing domain, the stem domain, and the clip domain (Fig 2E). The clip domain provides an attachment site for the connector and interacts with the terminase motor during DNA packaging, as in phages P22 [43] and HK97 [44].

The connector (Figs 1A and 2D), attached below the portal, comprises three components: a dodecameric adaptor, a hexameric stopper, and a hexameric terminator, and exhibits structural conservation across the majority of siphophages [45–49] and myophages [20,23,50]. The adaptor protein gp126 consists of an α-helix domain, a β-sheet domain, and a C-arm (Fig 2E). The C-terminus of each adaptor gp126 extends upward and interacts closely with the outer surface of the clip domain of the portal protein, forming an augmented four-stranded β-sheet (Fig 2D). The hexameric stopper interacts with the 24-stranded β-barrel situated at the bottom of the adaptor, thereby facilitating the transition from 12-fold to 6-fold symmetry (Fig 2D). Each stopper protein gp128 adopts a typical β-barrel fold (Fig 2E). The tail terminator contains six copies of the protein gp129, each with an N-terminal domain and a β-hairpin domain (Fig 2D, 2E).

Phi92 possesses a contractile tail with a height of 960 Å (Figs 1A and 2F), composed of 24 stacked hexameric rings of tail tube protein gp131 (inner) and sheath protein gp130 (outer). The tail tube protein gp131, structurally conserved among siphophages [51,52], myophages [18,53], and CISs [54,55], consists of a β-sandwich domain flanked by an α-helix and an extended β-hairpin (Fig 2G). The tail sheath protein gp130 can be divided into an N-terminal arm, domain I, domain II, domain III, and a C-terminal arm (Fig 2G), and exhibits a high structural similarity to the sheath proteins of other myophages [15,23,56] and CISs [54,55]. Domain I of the last sheath ring interacts with the C-terminal β-hairpin domain of terminator protein gp129 via an interlaced four-stranded β-sheet (Fig 2F). Within a sheath ring, the N-arm of one gp130 and the C-arm of its neighbor, in conjunction with a two-stranded β-sheet from the domain I of a monomer in the adjacent ring, form a four-stranded β-sheet handshake interaction that bridges and stabilizes neighboring sheath rings (Fig 2F). The interactions between the first sheath ring and the sheath initiator protein are described below. In addition, each tail tube protein monomer interacts with its neighboring sheath monomer through the cation-π interaction, hydrogen bond, and salt bridge (Fig 2F).

### Structure of the baseplate in the extended phi92

**Fig 3:**
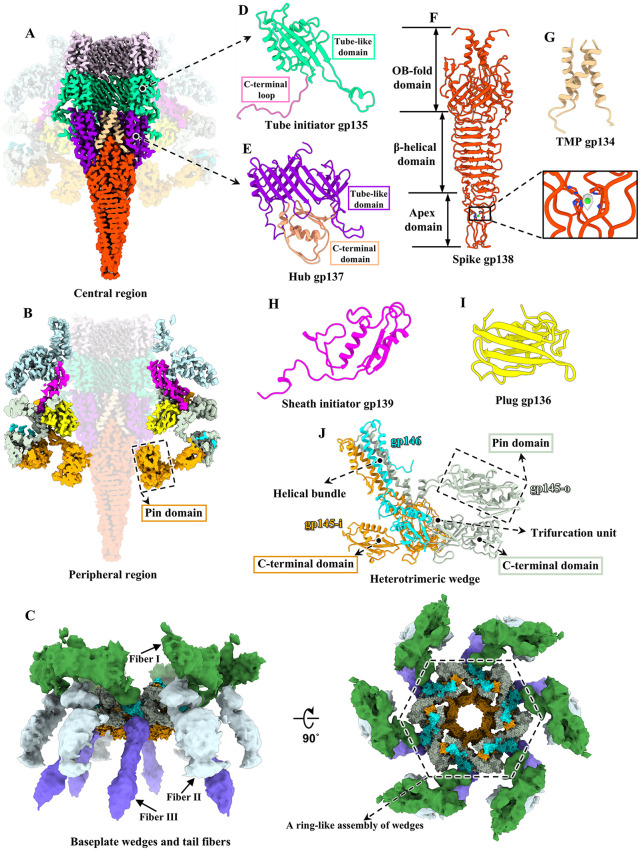
Structures of the baseplate in extended phi92. **(A, B)** Cut-open views of the density map of the central and peripheral regions. The color coding is identical to that used in Fig 1A. The peripheral region in panel A and the central regions in panel B are shown in transparency, respectively. **(C)** Side (left) and bottom (right) views of both the ribbon models (surface representation) of the baseplate wedges and the density maps of the tail fibers. **(D, E)** Ribbon models of the tube initiator protein gp135 and hub protein gp137, colored according to their domains. **(F, G)** Ribbon models of trimeric spike protein gp138 (F) and the trimeric C-terminus of TMP gp134 **(G)**. The inset shows the zoomed-in view of the density map of the Fe ion (green dot). **(H, I)** Ribbon models of the sheath initiator protein gp139 (H) and plug protein gp136 **(I)**. **(J)** Ribbon models of gp145-i (orange), gp145-o (gray) and gp146 (cyan) in a heterotrimeric wedge.

The baseplate of phi92 shares a conserved structural architecture with other myophages [15,20,21,23] and CISs [54,55]. It can be divided into central and peripheral regions (Fig 3A, 3B). The central region contains a hexameric tube initiator (gp135), a trimeric hub (gp137), a trimeric spike (gp138), and the C-terminus of the trimeric TMP (gp134). The peripheral region consists of six heterotrimeric wedges (gp145-gp146), a six-fold sheath initiator (gp139), a six-fold plug (gp136), and six copies each of fiber I (gp143), fiber II, and fiber III (gp147) (Fig 3B, 3C).

In the central region of the baseplate, the tube initiator protein gp135 contains a tube-like domain and a flexible C-terminal loop (Fig 3D), initiating the assembly of tail tube. Hub protein gp137 comprises a tube-like domain and a C-terminal domain (Fig 3E). The tube-like domains of gp135 and gp137 exhibit folds similar to tube protein gp131, forming six-fold and pseudo-six-fold symmetric tube rings (S7 Fig), as seen in Mu [15], phiTE [17] and Pam3 [20]. Spike protein gp138 contains an oligosaccharide-binding (OB-fold) domain, a β‑helical domain, and an apex domain (Fig 3F), structurally similar to that of phage P2 [27]. Notably, the apex domain of the trimeric gp138 coordinates a ferric ion via three H × H double-histidine motifs (Fig 3F), stabilizing the tip structure for piercing the host cell membrane during infection. Our cryo‑EM structure of gp138 is consistent with the reported crystal structure [27]. In addition, the C-terminal domain (residues 631–656) of the trimeric TMP gp134 was resolved, which is situated in the baseplate interior (Fig 3A, 3G).

In the peripheral region, the six-fold sheath initiator sits at the top of the wedge, and each sheath initiator protein gp139 shares structural homology with the I domain of the tail sheath protein gp130 (Figs 3H and S8A). Similar to the inter-ring handshake mechanism, gp139 forms a four-stranded β-sheet handshake interface with the N-arm of one sheath protein and the C-arm of a neighbor in the first sheath ring (S8B Fig), firmly anchoring the baseplate to the sheath. The sheath initiator protein is critical for sheath assembly and contraction, and is highly conserved across CISs [57,58] and other myophages [19,22]. Six copies of plug protein gp136 are arranged around the hub periphery, serving as a docking site for the wedge (Fig 3B). Each plug protein gp136 adopts an Ig-like fold structure (Fig 3I), a conserved structural and functional feature among myophages. The wedge is composed of six heterotrimers assembling into a ring-like structure (Fig 3C). Each heterotrimer contains one gp145 monomer and two conformational variants of the gp146 monomer, referred to as gp146-i located at “inner” portion of the wedge ring and gp146-o located at “outer” portion of the wedge ring (Fig 3J). The heterotrimer adopts a radial orientation: gp145 and gp146-o face the baseplate periphery (Fig 3J), while the pin domain of gp146-i faces the baseplate center, forming part of the inner interface adjacent to the central spike and hub (Fig 3B). The N-terminal segment of each monomer assembles into a core helical bundle, while their C-terminal domains adopt a canonical trifurcated fold (Fig 3J).

### Structures of the three fibers in the extended phi92

The phi92 phage assembles three distinct types of tail fibers, each comprising six trimers attached to the baseplate wedge (Fig 3C). Fiber I adopts an L-shaped conformation and connects to the upper end of the C-terminal domain of wedge protein gp145 (Fig 3C). Fiber II connects to the base of fiber I and extends downward at ~30° (Figs 3C and S3A). Fiber III connects to the bottom of the C-terminal domain of gp145 and points downward at ~37° (Figs 3C and S3A). In comparison with resolution previously reported [9,26], local reconstruction of fiber III using cryoSPARC improved its resolution to approximately 7 Å (S9A Fig). Although insufficient for atomic modeling, the N-terminal domain of the AlphaFold3-predicted gp147 trimer fits well with the density map, supporting the identification of fiber III as gp147 (S9B–S9C Fig). The predicted gp147 trimer exhibits structural similarity to the trimeric fibers of phages Mu [15] and Milano [18] (S9D Fig). Attempts to resolve fibers I and II using the same approach did not improve resolution; however, we provided detailed hypotheses regarding their structures and functions.

Capsular polysaccharides on the bacterial surface resist phage infection by acting as a physical barrier. Phage phi92 must penetrate a thick polysaccharide capsule to infect strains like *E.coli* K1 and K92 [12,24], indicating that capsule degradation is a crucial step for phi92 to initiate infection. These capsules consist mainly of polysialic acid with α2,8- or α2,9-linkages [59,60]. Biochemical experiments have confirmed that phi92 encodes endosialidase gp143, a bifunctional enzyme hydrolyzing both types of polysialic acid [12], explaining its broad host range. Although gp143 density was not discernible in our cryo-EM map, sequence analysis reveals that gp143 shares 51%, 52%, and 54% identity with endosialidases of phages K1E [61], K1-5 [62], and K1F [63], respectively (S10A Fig). AlphaFold3 trimeric predictions of endosialidases of phages K1E, K1-5 and K1F (S10A Fig) and the crystal structure of K1F endosialidase [64] reveal a characteristic mushroom-shaped conformation and a putative active site, similar to the gp143 structure in phi92, suggesting that these proteins share a common evolutionary origin and functional conservation. To date, only low-resolution tail fiber structures of K1E and K1-5 have been reported [65]. Their fibers share an L-shape architecture, with an arm-like scaffold protein binding an endosialidase at the distal end. The density of fiber I of phi92 is conformationally similar to the tail fibers of K1E and K1-5 (S10B Fig). The predicted trimeric structure of phi92 gp143 fits the density corresponding to the catalytic domain at the fiber tip (S10C Fig). Based on these comparisons, we speculate that the distal region of fiber I corresponds to the trimeric gp143 of phi92.

Although the extremely low resolution of the fiber II density in the extended baseplate precluded atomic model building, fiber II plays a pivotal role in the initial infection stages of phage phi92. Therefore, we employed AlphaFold3 to predict the structures of all proteins encoded by the phi92 genome and systematically screened them for candidates capable of adopting tail fiber-like architectures. Based on structural topology comparisons with tail fibers or spikes from other phages [66,67], we identified four putative tail fiber proteins, gp15, gp140, gp150, and gp151, with the exception of gp143 and gp147 (S11 Fig). In particular, the previous report [25] indicates gp150’s potential role in degrading the colanic acid layer on the bacterial surface, thereby facilitating the phage in breaching the exopolysaccharide barrier. We performed a structural homology search of gp15, gp140, and gp151 using the Dali server [68]. The results revealed that gp15 lacks homologous structures; Gp140 and gp151 share high homology with the head spike protein gp52 of phage Pa223 [69], but the function of these proteins remains uncharacterized. However, the AlphaFold3-predicted structures of these candidate proteins did not fit well into the low-resolution density map of fiber II. Notably, these structures also showed poor fitting to the previously reported density map of the corresponding fiber in the extended state of phi92 [9], where gp150 was speculated to be fiber II. Currently, the structural and genomic prediction of fiber II remains a significant challenge.

### Structural changes of the head-tail complex in the extended and contracted states

**Fig 4:**
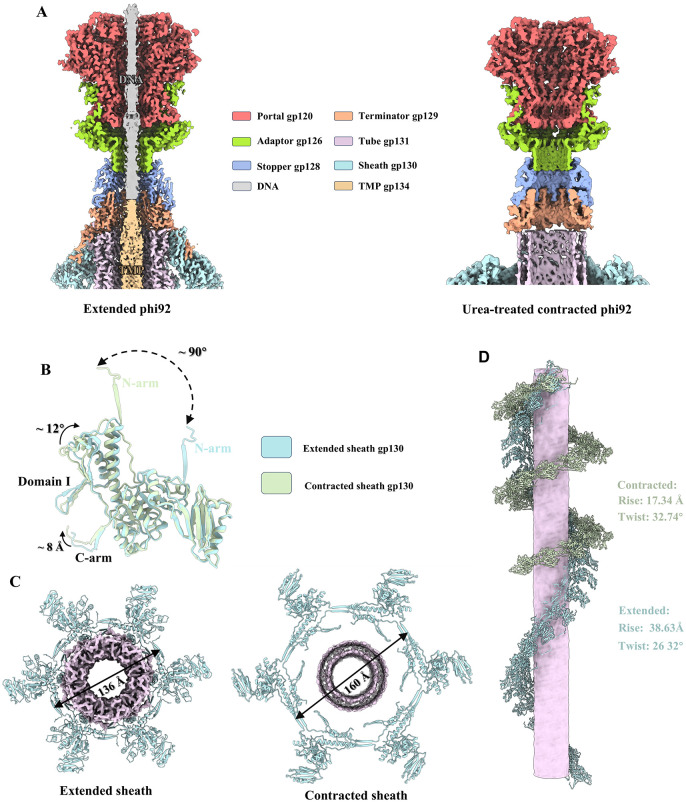
Structural changes of the portal-connector-tail complex of phi92 in the extended and contracted states. **(A**) Cut-open views of the density maps of the portal-connector-tail complex in extended phi92 (left) and urea-treated contracted phi92 (right). The color coding is identical to that used in Fig 1A. **(B**) Superimposition of the sheath monomers in the extended and contracted states showing the conformation changes. **(C**) Top views of the conformational changes of the tail from the extended to the contracted states. **(D**) Ribbon models of all sheath monomers in one helical pitch of extended (light blue) and contracted (dark green) states along tail tube (pink).

Urea treatment triggers conformational transition from the extended to contracted state, accompanied by significant changes in the baseplate and tail sheath, leading to TMP and genomic DNA release (Fig 4A) and yielding a contracted phi92 particle with an empty head, a contracted sheath, and an intact baseplate (Figs 1B and S5A).

In both states, the structures of the head, the portal, and the head-to-tail connector remain nearly unchanged, as observed in most siphophages [70] and myophages [15,19,23]. Notably, density for the C-terminal β-hairpin of the terminator protein gp129 was not resolved in the contracted state (Fig 4A). Based on terminator conformational changes in other myophages like E217 [23], Mu [15], and XM1 [19], we hypothesize that the flexible C-terminal β-hairpin domain of gp129 undergoes similar conformational rearrangement to maintain connection with the tail sheath during contraction. Structural analysis shows tube protein gp131 remains largely unchanged, while sheath protein gp130 undergoes substantial tertiary and quaternary rearrangements. At the tertiary structure level, structural superposition shows the N‑arm rotates ~100°, the C‑arm shifts ~8 Å, and domain I rotates ~12° (Fig 4B), indicating each sheath monomer undergoes rigid‑body rotation mediated by these regions during contraction. At the quaternary structure level, all sheath subunits remain connected via the four-stranded β-sheet handshake interaction (Figs 2F and S12), but the diameter of the sheath ring increases from 136 Å to 160 Å, disrupting the sheath-tube interface (Fig 4C). Helical twist and rise of the sheath change from 26.32° and 38.63 Å (extended) to 32.74° and 17.34 Å (contracted) (Fig 4D), resulting in a tighter sheath packing and exposure of ~460 Å of the rigid tail tube (Fig 1C). This contraction mechanism is conserved in myophages E217 [23] and Mu [15], and CISs like R-type pyocins [55] and PVCs [54]. Specifically, contraction transitions the tail sheath from a high-energy extended state to a low-energy contracted state, and the released energy drives tail tube penetration into the host cell membrane for genome delivery [13,71]. This contraction propagates wave-like along the tail axis toward the connector [72–74].

### Structural changes of the baseplate in the extended and contracted states

Urea-induced tail contraction in myophage phi92 causes the dissociation of the baseplate peripheral region from the central region (Fig 1C). High-resolution structure of the contracted baseplate central region was not obtained, so its protein composition remains unconfirmed. Density maps of phi92 baseplate show plug and spike dissociate from the tail tip (Fig 1C, 1D), consistent with phage E217 [23], suggesting conserved functions: the plug acts as a wedge docking site and may disengage upon wedge conformational change; the spike likely mediates the host outer membrane penetration and then dissociates to create a conduit for the TMP and genome release.

Structural change in the baseplate periphery during tail contraction is critical for stabilizing the fully contracted conformation of the tail apparatus. Structural comparisons between the extended and contracted states (Fig 1A, 1C) show that fiber I position remains essentially unchanged, while fiber III reorients from a downward-tilted conformation (extended) to a downward orientation perpendicular to the tail axis (contracted). A similar conformational change occurs in T4 short fiber during T4 contraction [22], suggesting fiber III rearrangement may transmit the contraction signal to the wedge. Fiber II was not observed in the contracted baseplate. Similarly, in phage A511, partial density of one type of its fibers was missing due to the urea-induced tail contraction [73]. Therefore, it is possible that the urea treatment during the preparation of the contracted sample severely disrupted the local structure of fiber II or its binding stability with the baseplate, causing it to detach from the tail and resulting in the subsequent loss of its signal. Morphologically, fiber II appears short, thick, and rigid, with structural features that likely share infection dynamics similar to the reported P22 gp9 [75] and Sf6 gp14 [76,77]. Structures of this type undergo subtle conformational changes and exhibit no obvious swinging movements during infection.

**Fig 5:**
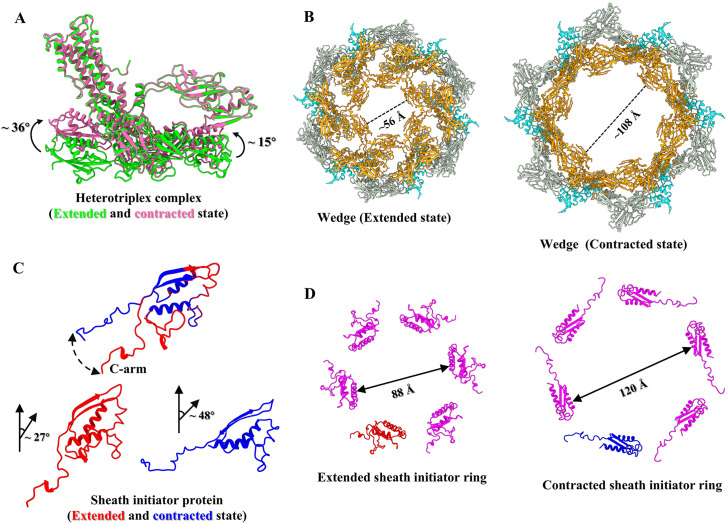
Structural changes of the baseplate in extended and contracted states. **(A)** Superimposition of a heterotriplex complex in the extended and contracted states. **(B)** Top view of the conformational changes from the extended wedge (right) to the contracted wedge (left). **(C)** Superimposition (top) and structural comparison (bottom) of the sheath initiator protein in the extended and contracted states. **(D)** Top view of the conformational change of the sheath initiator ring from extended (right) to contracted (left) states. All monomers in the rings are colored magenta, except for one monomer (colored red) from the extended ring and one monomer (colored blue) from the contracted ring.

Comparison of the heterotrimeric wedge structures in the extended and contracted states reveals that the core helical bundle remains largely unchanged, maintaining trimeric unit stability. However, the C-terminal domains of gp146-i and gp146-o undergo significant conformational changes, rotating approximately 36° clockwise and 15° counterclockwise, respectively (Figs 3J and 5A). In the extended baseplate, the C-terminal domains of gp146-i and gp146-o from adjacent heterotrimeric wedges form a dimerization interface via a handshaking interaction (S13A Fig). In the contracted baseplate, they move apart, transitioning the dimerization interface into a fingertip-like interaction (S13A Fig). These changes expand the wedge ring diameter from 56 Å to 108 Å (Fig 5B), accompanied by tail tip release. Although wedge rearrangements are conserved across CISs [57,78] and myophages [22,23], specific conformation details vary.

Sheath initiator protein gp139, though low in molecular weight, is crucial for structural integrity between the baseplate periphery and contractile tail sheath (S13B Fig). Comparison shows its C-terminal arm undergoes significant conformational change during contraction (Fig 5C), cooperatively facilitating subunit expansion and rotation. Notably, the gp139 C-terminus interacts closely with the baseplate wedge (S13C Fig). Thus, wedge ring expansion drives the expansion of the hexameric sheath initiator ring, increasing its diameter from 88 Å to 120 Å (Fig 5D). In addition, protein gp139 maintains strong interaction with the first sheath ring in the contracted state, forming a reinforced four-stranded β-sheet handshake structure (S12D Fig). The inclination angle of the handshake structure increases from 27° (extended) to 48° (contracted) (Fig 5C). These changes in gp139 trigger structural rearrangements in the first sheath layer, propagating wave-like upward through successive layers, ultimately driving global sheath contraction.

## Discussion

In this study, we resolved the intact structures of myophage phi92 in both extended and contracted states using cryo-EM and built atomic models from head to baseplate. Comparisons reveal that the structures of the head, portal, connector, and tail tube remain largely unchanged, with the exception of a conformational rearrangement in the β-hairpin of the terminator protein gp129 accommodating tail sheath contraction. The tail sheath undergoes dramatic compaction and contraction, exposing ~460 Å of the rigid tail tube. The conformational changes in phi92 from the head to the tail sheath are highly similar to those reported for other myophages [15,19,23,74] and CISs [55,57,58,78,79], suggesting a conserved contraction mechanism except for the baseplate.

Comparative analyses of the baseplates of myophages phi92, E217 [23], Mu [15], Milano [18], and phiTE [17] in the extended state reveal that central region proteins (hub, tube initiator, spike, and plug) are relatively conserved. Notably, some phages encode additional tube-linking proteins (e.g., gp37/gp38 in phage E217) or multifunctional proteins (e.g., gp34 in phage Mu, and gp25 in phage Milano) that integrate the tube initiator and plug functions. The baseplate periphery comprises six wedges, each attached to one or more sets of tail fibers, and the sheath initiator. However, recent studies on baseplate conformational changes have focused on E217 [23], and the trigger mechanism for tail contraction remains unclear. Given the structural similarity among known baseplates, we compared the conformational changes in E217 and phi92 baseplates to elucidate the contraction trigger mechanism.

As observed in both phages E217 [23] and phi92, irreversible tail fiber binding to the host induces substantial structural rearrangements in the baseplate periphery. Myophages tail fiber composition varies with host specificity [7]. E217 has one fiber set, and its six fibers alternate upward and downward conformations in the extended state, then all swing downward to bind host O-antigen in the contracted state, transmitting a signal to the wedge and triggering the conformational change in the baseplate. Phi92, possessing three distinct fiber types, may represent an evolved mechanism for efficient adsorption, broader host recognition, or enhanced host search and infection. Considering phi92’s ability to infect both capsulated and non-capsulated bacteria, we propose hypotheses regarding the functions of each fiber. Fiber I gp143 is likely primarily dedicated to degrading the bacterial capsule, engaging in reversible interactions with capsular polysaccharides on the cell surface. Fiber III gp147, which extends downward parallel to the tail axis and undergoes drastic conformational changes during infection, likely functions as the irreversible adsorption, mediating tight binding to bacterial receptors, similar to gp12 of T4 [22] and gp16 of SU10 [80]. Given the distinct proposed roles for fibers I and III, together with the infection mechanisms of other phages equipped with two types of tail fibers (e.g., phages T4 [81] and SU10 [80]), where one set of fibers typically mediates reversible adsorption and the other is responsible for irreversible binding, we further hypothesize that fiber II functions as a receptor binding protein that mediates the reversible adsorption of phi92 to surface receptors on non-capsulated bacteria.

Upon contraction, the six-fold symmetric wedge ring expands outward and moves away from the baseplate center in both E217 and phi92, while the peripheral contact mode transitions from a handshake to a fingertip. However, heterotrimeric wedge subunit analysis reveals differences: in E217, the pin domain of wedge protein gp44-a undergoes a dramatic conformational change (angle with C-terminal domain decreases from 70° to 35°), while in phi92, the pin domain of gp146-o shows no substantial conformational change; instead, the C-terminal domains of both gp146-i and gp146-o rotate pronouncedly. Thus, baseplate wedge conformation dynamics may differ at the tertiary structure level across myophages. Furthermore, the sheath initiator maintains structural integrity and is critical for contraction. In E217, its conformational changes were ambiguous due to map resolution limits. In phi92, the C-terminal arm of gp139 undergoes significant rearrangements, facilitating hexamer expansion and rotation. The sheath initiator drives conformational changes in the first sheath layer, possibly serving as a key signal for global sheath contraction. Similar conformational changes in the sheath initiator across myophages [19,22] and CISs [57,78] suggest a conserved mechanistic role. In summary, the baseplate periphery exhibits distinct conformational transition patterns during tail contraction in different myophages. Signal transmission by different tail fiber types induces wedge rearrangement; wedge expansion modes may vary, triggering sheath initiator conformational change and ultimately initiating tail contraction.

Polysialic acid capsules are critical virulence factors in pathogens like *E.coli* K1, *Neisseria meningitidis* and *Streptococcus agalactiae* [5]. By forming a dense physical barrier, these capsules partially impede the penetration of antibiotic molecules and facilitate evasion of phagocytosis and clearance by host immune cells, contributing to antimicrobial resistance and immune surveillance. Based on the structural analyses of phi92 and insights from other myophages [10,15,18] and CISs [51,53,54,66], we propose a detailed infection model for a myophage infecting capsulated bacteria. The process begins with capsule hydrolysis by fiber I, allowing phage penetration (S14A Fig). Fiber III then undergoes conformational change upon irreversible binding to outer membrane receptors, stably anchoring the baseplate to the bacteria (S14B Fig). Fiber III transmits the recognition signal to the wedge, triggering its rearrangements (S14C Fig). The contact mode transitions from a handshake to a fingertip, driving wedge expansion and sheath initiator conformational change (S14C Fig). The sheath initiator transmits the contraction signal to the sheath, causing wave-like propagation toward the connector and irreversible sheath contraction for genome injection (S14D Fig). For a myophage infecting non-encapsulated bacteria, fiber II may facilitate reversible adsorption (S15A Fig). Subsequently, as described above, a cascade of events ensues sequentially: Fiber III-mediated irreversible binding, rearrangement and signal propagation of baseplate wedge and sheath initiator proteins, tail sheath contraction, and genome injection (S15B-S15D Fig). This study reveals the cooperative advantage provided by multiple tail fibers in efficiently recognizing and penetrating encapsulated bacteria, and elucidates the molecular details of a universal mechanism by which conformational changes in baseplates trigger tail contraction. These findings provide a critical structural blueprint and molecular foundation for the future rational design, engineering, and optimized application of myophage-based phage therapies targeting capsulated drug-resistant bacteria.

## Materials and Methods

1. Production and purification of phage phi92

*E. coli* strain BL21 was cultivated in Luria-Bertani (LB) broth (10 g/L tryptone, 5 g/L yeast extract, and 10 g/L NaCl) at 37°C for 4 hours. Phage phi92 was next added to the bacterial culture at 37°C for 8 hours. After complete bacterial lysis, the cell debris was removed by centrifugation at 8000 × g for 30 min at 8 °C. Phage particles in the supernatant were precipitated with 1 M NaCl and 10% PEG8000, and cultured at 4 °C overnight. The phage precipitate was recovered by centrifugation at 4,500 rpm for 25 min and resuspended in TNM buffer (50 mM NaCl, 10 mM Tris-HCl, 5 mM MgCl2, and pH 7.4). Subsequently, phage particles were purified by ultracentrifugation through a CsCl step gradient (1.4, 1.5, and 1.7 g/mL) at 135,000 × g for 2 hours at 8 °C. The phage bands were dialyzed in TNM buffer at 4°C overnight to remove CsCl. Subsequently, the sample was diluted 10-fold in 3 M urea and then incubated in TNM buffer at 37 °C for 2 h to generate phi92 phage particles with contracted tails. After ultrafiltration to remove urea, phage tail contraction was detected by negative staining and cryo‑EM. Finally, the extended and contracted phi92 phage preparations were stored in ice water for cryo-EM sample processing.

2. Data acquisition and icosahedral reconstruction

The extended phages aliquot (3 µL) and contracted phages aliquot (3 µL) samples were applied separately to a Quantifoil R2/1 copper grid, which had been glow-discharged for 30 seconds at 20 mA. The grids were loaded into an FEI Vitrobot set to 8°C and 100% relative humidity with a 4.0 s blotting time. After blotting, the grids were rapidly vitrified by plunging into liquid ethane and subsequently stored in liquid nitrogen. Cryo-EM data were collected on a 300 kV Titan Krios G3i electron microscope, equipped with a K3 summit direct electron detector. Data for extended and contracted phage samples were collected automatically using the FEI EPU v2.12 software at magnifications of 75,000× and 59,000 × , which correspond to pixel sizes of 1.1 Å and 1.4 Å, respectively. A total of 7,893 and 5,617 movies, saved in TIFF format, were collected for the extended and contracted phages, respectively, with corresponding total electron doses of approximately 32 and 30 e^−^/Å^2^. The multi-frame movies of extended and contracted phages were drift-corrected using MotionCor2 [82]. Defocus and astigmatism values for each micrograph were estimated using GCTF software [83]. The extended and contracted particles were manually selected for analysis using the software ETHAN [84]. The head structures of the extended and contracted phi92 particles were determined using our own software [31], which is based on the common-line algorithm [85,86]. The head five-fold region of the extended phi92 was refined to a resolution of 3.9 Å using the local reconstruction method [34].

3. Symmetry-mismatch and local reconstruction

The asymmetric structure of myophage Mu [15], filtered to a resolution of 60 Å, was used as an initial model. Using the symmetry‑mismatch reconstruction method [32,35], the three‑dimensional asymmetric structure of the head–connector complex in the extended phi92 was obtained at low resolution. The reconstruction steps were as follows: (1) For each particle image, we determined the asymmetric orientation by searching the 60 equivalent icosahedral orientations based on an initial model. (2) A new asymmetric structure was reconstructed from the latest orientations, with no symmetry imposed. (3) The aforementioned steps were repeated iteratively until the orientations of all particle images converged. The capsid-portal, portal-adaptor and terminator-tail structures of the extended phi92 were reconstructed at 4.5 Å, 3.3 Å and 3.5 Å resolutions, respectively, by imposing C1, C12 and C6 symmetries. The portal-adaptor and terminator–tail structures of the contracted phi92 were resolved using the same method at resolutions of 4.5 Å and 4.9 Å, respectively. The helical parameters of the extended (rise 38.63 Å and twist 26.32°) and contracted (rise 17.34 Å and twist 32.74°) sheaths were obtained using the HI3D software [87].

Using the RELION 3.1.4 software [36], the local structures of the baseplate of the extended and contracted phi92 particles were reconstructed. A total of 32,356 particles of extended baseplate were manually selected with a box size of 360 × 360 pixels. Subsequently, poor particles were removed through multiple rounds of 2D and 3D classification. The extended baseplate was reconstructed to a resolution of 3.2 Å by imposing C3 symmetry. Using cryoSPARC 4.4.1 software [88], the particles were then symmetry-expanded six-fold and re-centered to the tail fiber. Following masking and refinement with C1 symmetry, the structure of fiber III was resolved at 7 Å. Furthermore, we selected a total of 9,796 particles of the contracted baseplate with a box size of 400 × 400 pixels. Using the same approach as described above, the contracted tail and baseplate structures were reconstructed at resolutions of 3.7 Å and 4.2 Å, respectively.

4. Model building and refinement

Based on the cryo-EM density maps of the extended phi92 with AlphaFold3 predictions [30], the atomic models of MCP gp124, CP gp123, portal protein gp120, adaptor protein gp126, stopper protein gp128, tail terminator protein gp129, sheath protein gp130, tube protein gp131, tube initiator protein gp135, sheath initiator protein gp139, the C-terminus of TMP gp134, hub protein gp137, spike protein gp138, plug protein gp136 and heterotriplex complex gp145-gp146 were built by using COOT software [89]. Based on the cryo-EM density maps of the contracted phi92, the atomic model of sheath protein gp130 was built. Additionally, we constructed backbone models of the initiator protein gp139 and the heterotrimeric complex gp145-gp146. All atomic models were further refined through real-space refinement in Phenix [90]. Refinement and validation statistics are listed in S1 Table.

## Supporting information

S1 Fig Cryo-EM images and Fourier shell correlation curves of two states of phi92. (A, B) Representative cryo-EM images of the extended (A) and contracted phi92 particles (B). (C) Left: Estimated structural resolutions of the capsid (bule), the baseplate (lime), the portal-adaptor (orange), the stopper-tail (yellow), portal-capsid (purple), and the tail fiber (light pink) in extended phi92. Right: Estimated structural resolutions of the capsid (magenta), the tail sheath (cyan), the baseplate (lime), portal-adaptor (purple), stopper-terminator (orange) in contracted phi92.
(TIF)

S2 Fig Local resolution maps of extended phi92 from the head to the baseplate. (TIF)

S3 Fig Quality of the cryo-EM density maps and atomic models from the head-tail in the extended phi92. (A) Cut-open view of the intact structure of extended phi92 with manually removed partial DNA. Color codes are identical to that used in Fig 1A. The inset shows the tilt angles of fibers II and III relative to the Z-axis. (B) Ribbon models of almost all protein components from the head-tail and density maps (transparency) of most components superimposed on their atomic models.
(TIF)

S4 Fig Local resolution maps of contracted phi92 from the head to the baseplate. (TIF)

S5 Fig Quality of the cryo-EM density maps and atomic models from the head-tail in the contracted phi92. (A) Cut-open view of the head-tail in the contracted phi92. Color codes are identical to that used in Fig 1B. (B) Ribbon models of all protein components from the sheath-baseplate and density maps (transparency) of gp130 and gp139 superimposed on their atomic models.
(TIF)

S6 Fig Structures of the MCP gp124 and CP gp123 in phi92. (A) Top view of ribbon models of the penton and hexon. Color codes are identical to that used in Fig 2A. (B) Top view of interactions along the three-fold axis, and the zoomed-in view of the MCP-CP interaction and density maps (transparency) of the MCP-CP superimposed on their atomic models. Three hexons are colored medium purple, green, and scarlet, respectively.
(TIF)

S7 Fig Structural comparisons of the tube initiator and hub proteins. (A) Structural comparisons of the tube-like domain (light green) among proteins gp131, gp135 and gp137 in extended phi92. (B) Bottom view of the ribbon models of the six-fold symmetric gp135 and pseudo-six-fold symmetric gp137. Color codes are identical to that used in Fig 1A.
(TIF)

S8 Fig Structures of the sheath initiator protein gp139 and sheath protein gp130 in phi92. (A) Structural comparisons of the similar domain (orange) between proteins gp130 and gp139 in extended phi92. (B) Top and side views of the ribbon models of the sheath initiator and sheath. The inset shows the zoomed-in view of the interactions between the sheath initiator and sheath, and density maps (transparency) of the sheath initiator and sheath superimposed on their atomic models. Color codes are identical to that used in Fig 1A.
(TIF)

S9 Fig Structure of fiber III gp147 in phi92. (A) Processing strategy of cryo-EM data from extended phi92 tail fiber. (B) Ribbon model of gp147 predicted by AlphaFold3. (C) Density map (transparency) of fiber III superimposed on the atomic model of gp147 modelled by AlphaFold3. (D) Structural comparisons between the ribbon models of phi92 gp147, Mu gp49 and phiTE gp236, predicted by AlphaFold3.
(TIF)

S10 Fig Structural comparisons of endosialidases between phi92 and other phages. (A) Comparison of structural similarity and sequence identity of endosialidases among phi92 and other phages. All structures of endosialidases in phi92 and other phages are predicted by AlphaFold3. The insets show the putative active sites of the endosialidases. (B) Structures of the fiber complexes of phages K1E and K1-5 (the structures are derived from the article J Mol Biol. 2007; 371:836–49), and the baseplate of phage phi92. The arm-like scaffold proteins and the endosialidases are highlighted by black and blue dashed boxes, respectively. (C) Density maps (transparency) of K1E tail (left, EMD-1333) and K1-5 tail (right, EMD-1335) superimposed on the atomic model of the endosialidases gp143 in phi92.
(TIF)

S11 Fig Structures of the putative tail fibers in phi92. (TIF)

S12 Fig Structure of the contracted sheath in phi92. The inset shows the zoomed-in view of the inter-ring and intra-ring interactions among contracted sheath monomers, and density maps (transparency) of the contracted sheath monomers superimposed on their atomic models.
(TIF)

S13 Fig Structure of the contracted baseplate in phi92. (A) Top view of the ribbon models from the extended (right) and the contracted (left) baseplate wedges. The top inset shows a zoomed-in view of the handshaking interaction between adjacent heterotriplexes. The bottom inset shows a zoomed-in view of the finger-like interaction between adjacent heterotriplexes. (B) Side view of the contracted baseplate in phi92. (C) Zoomed-in views of the box regions in panel B to show the interactions in the heterotriplex-initiator sheath. (D) Zoomed-in views of the box regions in panel B to show the interactions in initiator sheath-tail sheath, and density maps (transparency) of the sheath initiator-tail sheath superimposed on their atomic models. The color coding is identical to that used in Fig 1E.
(TIF)

S14 Fig Schematic diagram of phi92 contraction and DNA-ejection pathway during the infection of encapsulated bacteria. (A) Extended phi92 utilizes fiber I to degrade the bacterial capsular polysaccharide layer. The scissors represent the hydrolysis of the bacterial capsule by fiber I. (B) Fiber III irreversibly binds to receptor molecules on the host cell surface, undergoes conformational rearrangements, and transmits the contraction signal to the baseplate. Lightning represents the generation of the contraction signal. The closed lock represents the tight connection between the periphery and the interior of the baseplate. (C) The outward expansion of the baseplate wedges and the sheath initiator protein further triggers sheath contraction. The open lock represents the peripheral region of the baseplate moving away from its interior. (D) Finally, the sheath contraction drives the tail tube to pierce the outer membrane, forming a complete transmembrane channel in conjunction with the TMP that penetrates the inner membrane to enable genome release. The color coding is identical to that used in S3 Fig.
(TIF)

S15 Fig Schematic diagram of phi92 contraction and DNA-ejection pathway during the infection of non-encapsulated bacteria. (A) Extended phi92 utilizes fiber II to reversibly recognize receptors on the host cell surface. (B) Fiber III irreversibly binds to receptor molecules on the host cell surface, undergoes conformational rearrangements, and transmits the contraction signal to the baseplate. Lightning represents the generation of the contraction signal. The closed lock represents the tight connection between the periphery and the interior of the baseplate. (C) The outward expansion of the baseplate wedges and the sheath initiator protein further triggers sheath contraction. The open lock represents the peripheral region of the baseplate moving away from its interior. (D) Finally, the sheath contraction drives the tail tube to pierce the outer membrane, forming a complete transmembrane channel in conjunction with the TMP that penetrates the inner membrane to enable genome release. The color coding is identical to that used in S3A Fig.
(TIF)

S1 Table Refinement and model statistics of phi92. (XLSX)
